# IMPDH1, a prognostic biomarker and immunotherapy target that correlates with tumor immune microenvironment in pan-cancer and hepatocellular carcinoma

**DOI:** 10.3389/fimmu.2022.983490

**Published:** 2022-12-22

**Authors:** Chengdong Liu, Wanli Zhang, Xiaohan Zhou, Li Liu

**Affiliations:** ^1^ Department of Infectious Diseases, Nanfang Hospital, Southern Medical University, Guangzhou, Guangdong, China; ^2^ Department of Radiation Oncology, Nanfang Hospital, Southern Medical University, Guangzhou, Guangdong, China

**Keywords:** pan-cancer, IMPDH1, tumor immune microenvironment, immunotherapy, methylation, m-6-A, hepatocellular carcinoma (HCC)

## Abstract

**Backgrounds:**

IMPDH1, a rate-limiting enzyme in de novos synthesis of guanine nucleotides, plays an essential role in the growth and progression of certain tumors. However, there is still a lack of study on IMPDH1 evaluating its role in the tumor immune microenvironment, the potential mechanisms, and its potential as a promising tumor therapeutic target.

**Methods:**

The Cancer Genome Atlas (TCGA), Gene Expression Omnibus (GEO), Genotype-Tissue Expression (GTEx), TIMER2.0, KM-Plotter, University of Alabama at Birmingham Cancer data analysis Portal (UALCAN), cbioportal, The Human Protein Atlas (HPA), and Gene Expression Profiling Interactive Analysis 2 (GEPIA2) were used to perform the systematic analysis of IMPDH1, including mRNA expression, protein expression, prognostic value, Enrichment analysis, DNA methylation, immune cell infiltration in pan-cancer, Then, we conducted qRT-PCR and immunohistochemistry to analyze the expression level of IMPDH1 in cancer tissues and non-cancer tissues of patients with primary hepatocellular carcinoma (HCC), and performed the same verification at cellular level.

**Results:**

We discovered that IMPDH1 was highly expressed in a variety of tumors and was associated with poor prognosis. IMPDH1 not only had the potential as a tumor prognostic marker and therapeutic target, but also was closely related to immune cells, immune checkpoints and immune-related genes and pathways in the tumor immune microenvironment (TIME). Meanwhile, IMPDH1 expression influenced the efficacy and prognosis of tumor patients treated with immune checkpoint inhibitors.

**Conclusions:**

IMPDH1 may be as a potential combined target of immunotherapy.

## 1 Introduction

Malignant tumors threaten the life and health of all mankind. How to suppress and eliminate tumors has always been an eternal topic in human science. Finding specific biological markers and choosing appropriate treatment are essential. With the rapid development of precise oncology, cancer biomarkers play an increasingly important role as specific characteristics of tumor cells. It can not only help to diagnose new or recurrent diseases, judge the prognosis of diseases, but also predict the efficacy or toxicity of therapeutic drugs. At present, ESMO is committed to integrating cancer biomarkers into the core of future precision oncology and personalized cancer treatment (1). Different types of tumors have distinct treatment options. Hematoma are generally treated with chemotherapy, targeted therapy and immunotherapy (2–4). Solid tumors are mainly treated by surgical resection. However, due to various factors including the difficulty of tumor mass resection and distant metastasis, systemic therapy is often used to reduce clinical stage before surgery, prevent recurrence after surgery, or replace surgery in a variety of tumors (5–7). In the era of cancer precision therapy, important pathways and targets are particularly essential (8). In particular, immunotherapy as a first-line and second-line treatment for cancer has achieved remarkable achievement in recent years. At present, among some immune checkpoints used for tumor immune escape, the most far-reaching clinical applications are: programmed death receptor1/programmed death ligand1 (PD-1/PD-L1), cytotoxicity T lymphocyte antigen4 (CTAL4) (9). Nonetheless, low response rate and even drug resistance often occur during immunotherapy. Tumor suppressive immune microenvironment as a key factor influences the efficacy of immunotherapy. It is like HIF-1α regulates its antifungal immunological activity by regulating the glycolytic activity of dendritic cells and the release of some pro-inflammatory cytokines in the immune microenvironment (10). The number of CD8^+^T cells plays a particularly significant role in anti-tumor therapy and patient prognosis (11). In addition to the killing and memory functions of effector T cells, the phagocytosis of phagocytes such as macrophages and neutrophils also contributes to the progression of tumors. In innate immunity, high-density neutrophils (HDNs) of tumor-associated neutrophils (TANs) exhibit stronger anti-tumor effects, phagocytic activity and migration ability than low-density neutrophils (LDNs) (12).

In addition, tumor mutational burden, neoantigen burden, and pre-infiltrating T cells are all indicators of benefit from immune checkpoint inhibitor therapy (13–15), but the accumulation of mutational burden during mitosis also accumulates loss of methylation (16). The immune infiltration landscape of pancreatic cancer tumor microenvironment (TME) suggests that adenosine N6-methylation (m-6-A) methylation may affect the therapeutic effect of immune checkpoint inhibitors (17). In glioblastoma, by modulating the m-6-A modification pattern of key immune targets, it has a certain reversal effect on the immunosuppressive microenvironment (18). The modification of m-6-A is mainly accomplished by three major parts, and these m-6-A regulators are involved in many biological processes of tumorgenesis and development. ALKBH5 plays an immunomodulatory role in melanoma, colorectal cancer and other tumors, not only regulating Mct4/Slc16a3 molecules related to tumor survival, metastasis and metabolism, but also affecting the number of tumor-infiltrating Treg cells and bone marrow derived inhibitory cells (MDSC) (19). YTHDF1 may play different roles in different tumors. In a study of NSCLC, YTHDF1 and YTHDF2 were associated with a good prognosis of NSCLC patients, and down-regulation of YTHDF1 and YTHDF2 could up-regulate the expression of PD-L1 and reverse the tumor suppressive microenvironment (20). However, in gastric cancer, highly expressed YTHDF1 inhibits the immune microenvironment of gastric cancer by inducing the proliferation of tumor cells and inhibiting the infiltration of dendritic cells (21). In general, both positive and negative effects of m-6-A regulatory factor are closely related to tumor immunity.

Inosine Monophosphate Dehydrogenase 1 (IMPDH1), as a rate-limiting enzyme, catalyzes the synthesis of xanthine monophosphate (XMP) from 5’ -inosine monophosphate (IMP) in *de novo* synthesis of guanine nucleotides. Cell proliferation and high depletion of guanine nucleotides occur during lymphocyte antagonism against antigens. It can be found that IMPDH aggregates into cytosine structures during this process (22). Of the two subtypes, IMPDH1 is predominant in the retina, whereas IMPDH2 is constitutively expressed throughout the body. It has been reported that increased IMPDH1 expression promotes the formation of cytosine, thereby facilitating the consumption of guanine nucleotides, which is associated with uncontrolled proliferation, suggesting that IMPDH1 may influence the occurrence and development of malignant tumors (23), whereas there are few studies on the specific functions and potential mechanism of IMPDH1 affecting tumors, especially from the perspective of pan-cancer.

This study intends to explore the specific role of IMPDH1 in tumors to determine whether it is suitable as a key tumor biomarker, whether it can regulate tumor immunity, and whether it has an important impact on the immunotherapy of tumor patients. Therefore, we used bioinformatics analysis and some experiments to explore and verify the mRNA and protein expression levels of IMPDH1 in various tumors, as well as the relationship of IMPDH1 expression with tumor patients prognosis, estimate score, tumor purity, the infiltration of immune cells, and important immune molecules expression in tumor microenvironment. Particularly, we analyzed the correlation between IMPDH1 expression and treatment responsiveness, prognosis of cancer patients receiving immunotherapy. Then, we also analyzed the methylation level of IMPDH1 and the prognosis of patients related to the methylation level, revealing the potential mechanism of IMPDH1 overexpression in various tumors. Next, we mainly analyzed the correlation between IMPDH1 and immune cell infiltration score, multiple immune checkpoints in HCC microenvironment. We performed KEGG and GO analysis, and then evaluated the correlation between IMPDH1 and macrophages, neutrophils, which involved in top results of enrichment analysis. In order to explain the relationship between IMPDH1 and the immune microenvironment of HCC, we evaluated the correlation between IMPDH1 and m-6-A modified proteins, and also the relationship of these proteins with neutrophils, macrophages respectively.

## 2 Materials and methods

### 2.1 Data collection

RNA expression and clinical data from The Cancer Genome Atlas (TCGA) and Genotype-Tissue Expression (GTEx) were downloaded from the UCSC Xena database (https://xenabrowser.net/datapages/). The UALCAN (http://ualcan.path.uab.edu/analysis-prot.html) database was used to explore the protein levels of IMPDH1 in human tumors and normal tissues. The GEPIA2 (http://gepia2.cancer-pku.cn/#index) was used for conducting the correlation of IMPDH1 with m-6-A modified proteins. The GSE135222 dataset was downloaded from the Gene Expression Omnibus (GEO) database. IMvigor210 information was obtained from the R package “IMvigor210CoreBiologies”. Data for the CheckMate cohort was downloaded from the supplementary materials of a published work (PMID: 32472114) (24). Methylation (HM450) data were obtained from cbioportal (www.cbioportal.org). Partial tissue immunohistochemical images were downloaded from The Human Protein Atlas (https://www.proteinatlas.org/).

### 2.2 IMPDH1 prognostic analysis

Kaplan-Meier analysis was performed to evaluate the overall survival (OS), Progression Free Survival (PFS), Disease Free Survival (DFS), Disease Free Survival (DSS) of patients from TCGA cohort. Multivariate COX regression analysis and nomogram were also used to evaluate the prognosis of HCC patients in the TCGA database. Survival analysis of the GEO datasets was conducted using The R (version 4.1.1) and KM Plot (http://kmplot.com/analysis/index.php?p=background).

### 2.3 Tumor microenvironment characterization

Three TME phenotypes were defined, and the TME score was constructed using principal component analysis algorithms according to previous work (25).

### 2.4 Immune cell infiltration

We downloaded and analyzed the immune cell infiltration score of TCGA cohort from the ImmuCellAI database (http://bioinfo.life.hust.edu.cn/ImmuCellAI#!/), TIMER2 database (http://timer.cistrome.org/), and a previously published study (12, 26). For each TCGA tumor type, patients were divided into two groups with high and low IMPDH1 expression based on the median IMPDH1 expression level to compare the extent of immune cell infiltration.

### 2.5 Correlation and gene set enrichment analysis

The correlation analysis between IMPDH1 expression level and all protein-coding genes was performed using TCGA pan-cancer data, and Pearson’s correlation coefficient was further calculated. The top500 genes positively correlated with IMPDH1 (p<0.05) were subjected to GO and KEGG analysis using R package “clusterProfiler”.

### 2.6 Human tissue samples

The experiments involving human samples in this research were in accordance with the principles of the Helsinki Declaration and approved by the Institutional Review Committee of Nanfang Hospital, Southern Medical University, Guangdong Province, China (NFEC-201208-K3).The patients/participants provided their written informed consent to participate in this study. A total of 11 HCC tissues and paired non-cancer tissues were collected for qRT-PCR. HCC tissue and paired non-cancer tissue were collected for IHC.

### 2.7 Immunohistochemical

HCC patients tissues were obtained and subjected to formalin fixation, paraffin embedding, and sectioning. We used an Efficient immunohistochemical secondary antibody kit (Absin, CHN, Shanghai). The operation steps followed the previous study (27). The sections were stained with anti-IMPDH1 (Proteintech, USA, IL, 1:100 dilution).

### 2.8 Cell lines

MHCC-97H, HCC-LM3, Hep-G2, SK-Hep-1, Snu387, and MIHA were purchased form Procell (CHN, Wuhan). MHCC-97H, HCC-LM3, and Hep-G2 were cultured in DMEM medium (Gibico, USA, CA) obtained 10% FBS (ExCell Bio, CHN), and 1% penicillin and streptomycin (ExCell Bio, CHN). SK-Hep-1 was cultured in MEM medium (Biological Industrie, Israel) supplemented 10% FBS. Snu387, and MIHA were cultured in RPMI-1640 medium (Gibico, USA, CA) obtained 10% FBS and 1% penicillin and streptomycin. All cells cultured conditions were 37˚C and 5% CO2.

### 2.9 qRT-PCR

We conducted the extraction of total RNA, First Strand cDNA Synthesis (RT-PCR), and Real-time PCR, respectively by using TRIzol reagent (TaKaRa, JPN,TKY), PrimeScriptTM RT reagent kit with gDNA eraser (TaKaRa), and SYBR Green PCR kit (TaKaRa). Primers as follows:

ACTB:
Forward Primer: TCAAGATCATTGCTCCTCCTGA
Reverse Primer: CTCGTCATACTCCTGCTTGCTG
IMPDH1:
Forward Primer: CAGCAGGTGTGACGTTGAAAG
Reverse Primer: AGCTCATCGCAATCATTGACG

### 2.10 Statistical analyses

Data are presented as means ± standard error (SD). Differences between groups were analyzed using Student’s t-test or analysis of variance. Correlation between groups were conducted by using Pearson correlation analysis. Statistical analyses were performed using R version 4.1.1. p < 0.05 (two-tailed) was considered statistically significant.

## 3 Results

### 3.1 Expression analysis and verification of IMPDH1 in pan-cancer

**Figure 1:**
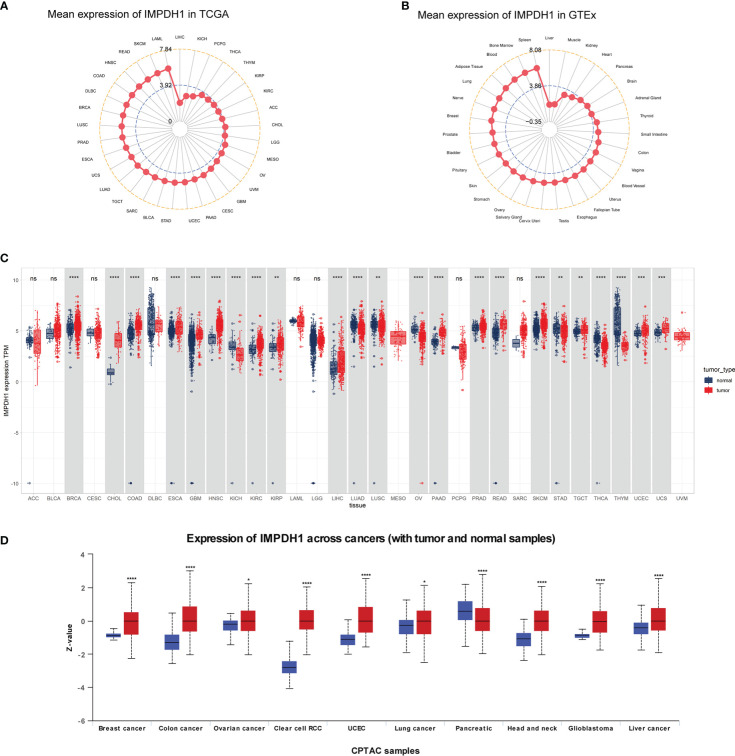
Pan-cancer mRNA and protein expression of IMPDH1. **(A)** Expression of IMPDH1 in tumor tissues from The Cancer Genome Atlas (TCGA) database. The location of the dot represents the mean value of IMPDH1 expression. **(B)** Expression of IMPDH1 in normal tissues from the Genotype-Tissue Expression (GTEx) database. The location of the dot represents the mean value of IMPDH1 expression. **(C)** Analysis of the mRNA expression of IMPDH1 in tumor tissues using TCGA database and in normal tissues using the GTEx and TCGA database. **(D)** Analysis of the protein expression of IMPDH1 in tumor and normal tissues through UALCAN database. *p < 0.05; **p < 0.01; ***p < 0.001 and ****p < 0.0001; ns, not significant.

At first time, we conducted expression analysis of IMPDH1 in 33/31 types of tumor-normal corresponding tissue from TCGA and GTEx database. The results indicated that IMPDH1 expressed highest in LAML, and lowest in LIHC (**Figure 1A**). For normal tissues, IMPDH1 expression was highest in spleen, and lowest in liver (**Figure 1B**). It was found that the expression of tumor tissue was higher than that of the corresponding normal tissue in majority of tumors (**Figure 1C**), as these 17 kinds of tumor: BRCA, CHOL, COAD, ESCA, GBM, HNSC, KIRC, KIRP, LIHC, LUSC, PAAD, PRAD, READ, SKCM, TGCT, UCEC, UCS. In contrast, there were still 6 tumors: KICH, LUAD, OV, STAD, THCA, THYM, expressed lower in tumors. Regrettably, the combinatory analysis was not able to conduct due to the lack of normal tissue data for MESO and UVM in GTEx. Furthermore, analysis of pan-cancer using the CPTAC datasets showed that the protein expression of IMPDH1 was also higher in tumor tissues than in normal tissues in breast cancer, colon cancer, ovarian cancer, clear cell renal cell carcinomas, uterine corpus endometrial carcinoma, lung cancer, pancreatic cancer, head and neck cancer, glioblastoma, and liver cancer (**Figure 1D**).

**Figure 2:**
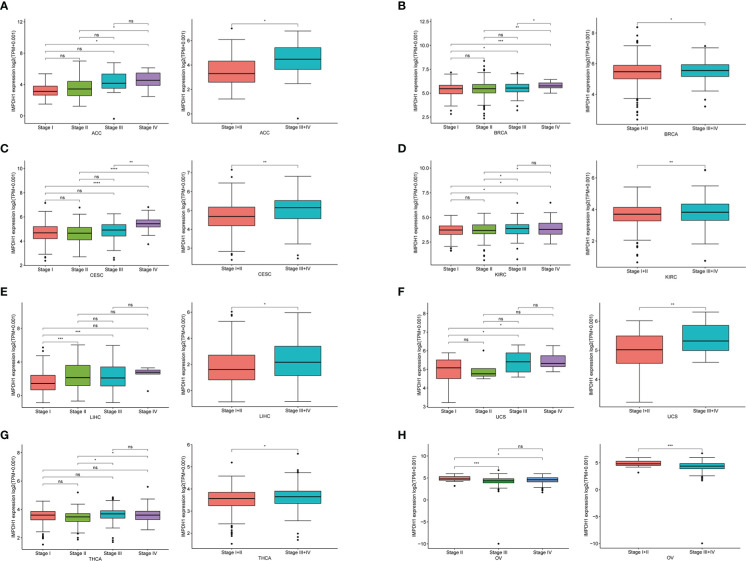
Correlation between the expression of IMPDH1 and TNM tumor stages. **(A-H)** Determination of pan-cancer IMPDH1 expression in four different TNM stages from TCGA database. The left panel represents stage I-IV and the right panel merges stage I with stage II, and merges stage III with stage IV. *p < 0.05; **p < 0.01; ***p < 0.001 and ****p < 0.0001; ns, not significant.

**Figure 3:**
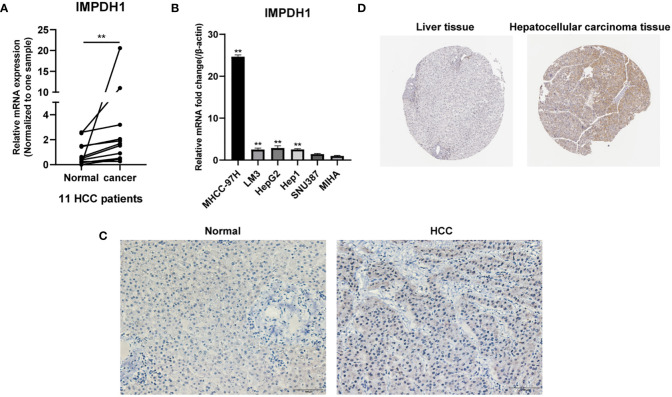
IMPDH1 is highly expressed in HCC tissues and cells. **(A)** The expression of IMPDH1 is higher in HCC patients tumor tissues than non-tumor tissues verified by qRT-PCR. **(B)** The expression of IMPDH1 is higher in HCC cell lines than hepatocytes verified by qRT-PCR. **(C)** The expression of IMPDH1 is higher in HCC patients tumor tissues than non-tumor tissues verified by IHC. **(D)** The expression of IMPDH1 is higher in LIHC patients tumor tissues than non-tumor tissues using The Human Protein Atlas database. **p < 0.01.

To determine the expression of IMPDH1 accurately, we analyzed the expression of IMPDH1 at different TNM stages manifesting IMPDH1 had higher expression in 7 types of tumors at advanced stages (stage 3/4): ACC, BRCA, CESC, KIRC, LIHC, UCS, THCA (**Figures 2A-G**). Unsurprisingly, IMPDH1 was expressed higher in OV at early stages (stage 1/2) (**Figure 2H**). In addition, we evaluated IMPDH1 expression in tumors and their paired adjacent tissues, which showed that in 14 kinds of tumors IMPDH1 were expressed higher in tumor tissue than paired adjacent tissue (**Figures S1A-N**), and just KICH had the opposite result (**Figure S1O**). We verified the IMPDH1 expression by using HCC patients’ paired tissues, HCC cell lines, and hepatic cell line. Compared with para-carcinoma tissues, IMPDH1 had a higher expression in HCC tissues, reflected by qRT-PCR result of 11 primary HCC patients (**Figure 3A**). Meanwhile the results of HCC and hepatic cells indicated IMPDH1 was highly expressed in HCC cells, especially in MHCC-97H, and the expression of IMPDH1 in each type of HCC cells was higher than that in hepatocytes (**Figure 3B**). We also verified the protein expression and subcellular localization of IMPDH1 in HCC patients cancer and paired non-cancer tissues by Immunohistochemistry (IHC) (**Figure 3C**), and supported by IHC results from “The Human Protein Atlas”, which were consistent with the results of bio-informatics analysis (**Figure 3D**).

### 3.2 High expression of IMPDH1 usually leads to poor prognosis of cancer patients

**Figure 4:**
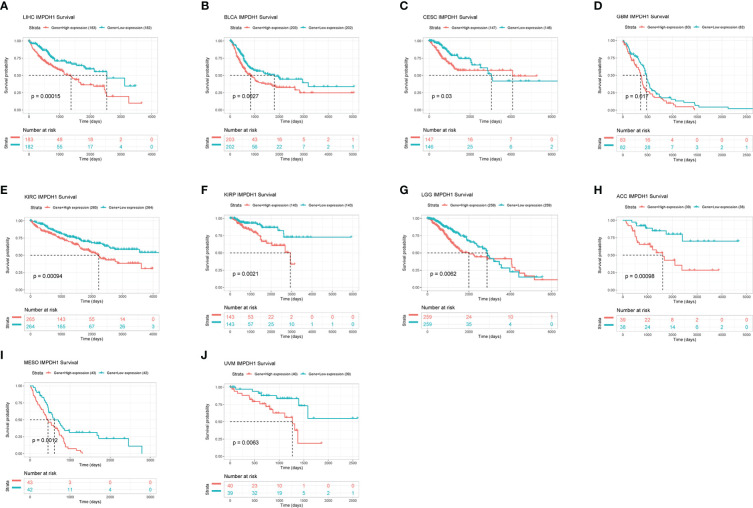
Association of IMPDH1 expression with overall survival. **(A-J)** Kaplan-Meier analysis of the correlation between the overall survival (OS) of cancer patients and IMPDH1 expression by using TCGA database. The median value of IMPDH1 expression in each tumor was taken as the cut-off value.

IMPDH1 was highly expressed in a variety of tumors. In order to explore whether the expression level of IMPDH1 correlated with patient survival, we assessed overall survival of cancer patients associated with IMPDH1 expression level in pan-cancer by using TCGA datasets. It could be found that 10 tumors had statistically significant results, indicating patients poor prognosis in high expression IMPDH1 in such tumors: LIHC, BLCA, CESC, GBM, KIRC, KIRP, LGG, MESO, UVM, ACC (**Figures 4A-J**) in TCGA database. Furthermore, we evaluated DFS, PFS, and DSS with TCGA database, and results suggested that tumor patients with high IMPDH1 expression usually had worse prognosis in LIHC, BLCA, CESC, KIRC KIRP, LGG, MESO, UVM (**Figures S2A-Q**). We also validated the association of IMPDH1 expression with OS and PFS in gastric cancer, lung cancer, liver cancer, and ovarian cancer patients using the GEO datasets through KM Plotter (**Figures S3A-H**). IMPDH1 expression was negatively correlated with survival in those cancer patients except for ovarian cancer. In addition, We also performed multivariate cox analysis of IMPDH1, age, gender, TNM stage and tumor grade in HCC patients. Results suggested that both IMPDH1 and TNM stage are independent risk factors for HCC patients. (**Figure S4A**). Therefore, we set up a scale score, and made a nomogram using IMPDH1 and TNM stage to predict the 1,3, and 5-year survival of HCC patients. (**Figure S4B**). By comparing the results of nomogram prediction and the observed situation, we found that the predicted 1,3, and 5-year survival were all distributed around the ideal value, and the 1,3, and 5-year survival of HCC patients could be accurately predicted by IMPDH1 expression and TNM stage (**Figure S4C**). Overall, IMPDH1 as an independent risk prognostic factor, could be used to predict a variety of cancer patients survival prognosis, such as HCC.

### 3.3 IMPDH1 is closely related to tumor immune microenvironment and influences the response of immunotherapy

**Figure 5:**
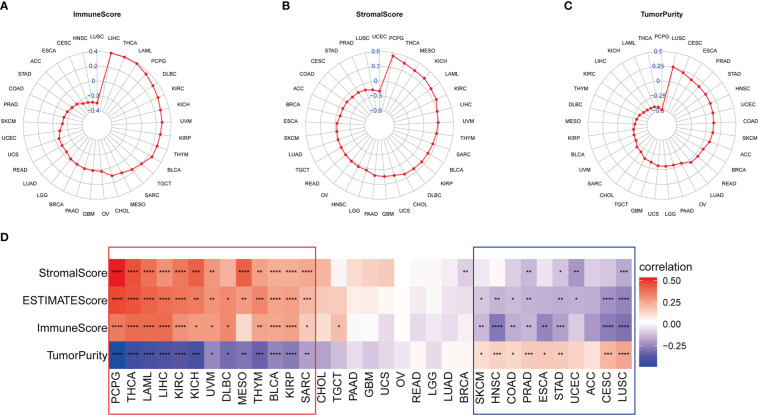
Association of IMPDH1 expression with tumor microenvironment in pan-cancer. **(A)** Analysis of the correlation between IMPDH1 expression and tumor Immune Score in pan-cancer. **(B)** Analysis of the correlation between IMPDH1 expression and tumor Stromal Score in pan-cancer. **(C)** Evaluation of the correlation between IMPDH1 expression and Tumor Purity in pan-cancer. **(D)** A heat map of the correlation between IMPDH1 expression and Immune Score, Stromal Score, Estimate Score, Tumor Purity. *p < 0.05; **p < 0.01; ***p < 0.001 and ****p < 0.0001.

To further explore the impact of IMPDH1 on tumor development, we first discussed the role of IMPDH1 in tumor immune microenvironment (TIME). It’s known that tumor cells, immune cells and stromal cells constituted the main cell types in tumor microenvironment (TME). The level of tumor purity score made a huge influence on tumor progress. Thus, we analyzed the correlation between IMPDH1 and Immune score, Stromal score, Tumor purity score at the same time (**Figures 5A-C**). As the results were shown that IMPDH1 was negatively correlated with tumor purity score and positively correlated with Immune score and Stromal score in 13 tumors: PCPG, THCA, LAML, LIHC, KIRC, KICH, UVM, DLBC, MESO, THYM, BLCA, KIRP, SARC. However, IMPDH1 was positively correlated with tumor purity score in 9 tumors: SKCM, HNSC, COAD, PRAD, ESCA, STAD, UCEC, CESC, LUSC. In two-thirds of pan-cancer tumors, the association of IMPDH1 with Tumor purity score and Estimate score was statistically significant (**Figure 5D**), which meant that IMPDH1 could influence tumor development by modifying tumor microenvironment in most tumors. However, due to the different types of tumors, the effect of IMPDH1 on tumor microenvironment scores also yielded opposite results, and how IMPDH1 affected tumor progression was still unclear, further work was needed to explore the content of this part.

**Figure 6:**
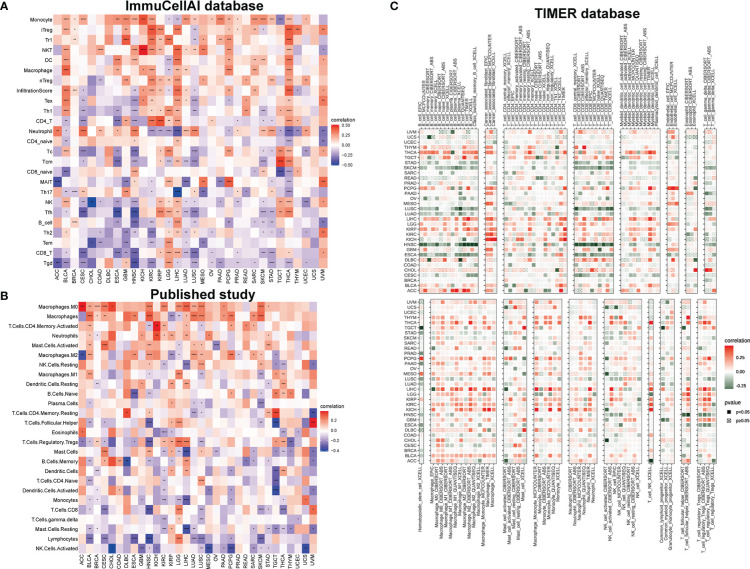
IMPDH1 is related to immune cell infiltration. **(A)** Association of IMPDH1 expression with immune cell infiltration by using the ImmuCellAI database. **(B)** Association of IMPDH1 expression with immune cell infiltration from published work. **(C)** Association of IMPDH1 expression with immune cell infiltration by using the TIMER2 database. *p < 0.05; **p < 0.01; ***p < 0.001 and ****p < 0.0001.

At present, immunotherapy gradually highlights its advantages in tumor treatment, promoting us to conduct analysis of the relationship between IMPDH1 with immune factors, such as immune cells and immune-related genes. At first time, we examined the correlation of IMPDH1 with tumor associated immune cells by using three different sources data, reflecting that IMPDH1 had a strong relationship with a large proportion of immune cells in most tumors. In the ImmuCellAI database, monocytes were confirmed to be the most common immune cells positively correlated with IMPDH1 in pan-cancer, while the most common negatively correlated cells were CD8^+^T and gamma delta T cells (**Figure 6A**). Meanwhile, we used published studies data (**Figure 6B**) and some data from TIMER database (**Figure 6C**) to verify the conclusion and the results were relatively in good agreement with ImmuCell AI database. In summary, significant correlation between monocytes, macrophages, CD8^+^T cell, gamma delta T cell and IMPDH1 in pan-cancer could be found in these databases.

**Figure 7:**
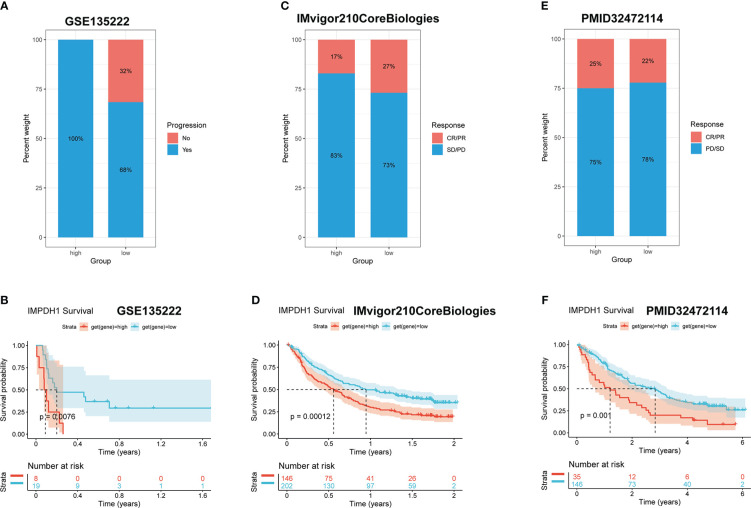
High expression of IMPDH1 is detrimental to the response and prognosis of tumor patients with immunotherapy. **(A)** Analysis of the immunotherapy response of non-small cell lung cancer patients related to IMPDH1 expression using GSE135222 dataset. **(B)** Evaluation of the progression-free survival of non-small cell lung cancer patients with anti-PD-1/anti-PD-L1 from GSE135222 dataset. **(C)** Analysis of the immunotherapy response of Urothelial carcinoma patients related to IMPDH1 expression using TCGA database. **(D)** Evaluation the overall survival of Urothelial carcinoma patients with anti-PD-1 from TCGA database. **(E)** Analysis of the immunotherapy response of Kidney renal clear cell carcinoma patients related to IMPDH1 expression using TCGA database. **(F)** Evaluation of the the overall survival of Kidney renal clear cell carcinoma patients with anti-PD-1 from TCGA database.

It has been preliminarily confirmed that IMPDH1 had a great influence on the infiltration of immune cells in the tumor immune microenvironment, and the immunotherapy effect was closely related to the immune checkpoint expressed on the surface of immune cells. To further understand whether the expression level of IMPDH1 is associated with the prognosis of tumor patients receiving immunotherapy, we analyzed the differences in therapeutic response and prognosis among IMPDH1 expression subgroups in three cohorts of patients receiving immunotherapy for non-small cell lung cancer (**Figures 7A, B**), urothelial cell carcinoma (**Figures 7C, D**), and renal clear cell carcinoma (**Figures 7E, F**). Consistent with our estimated results, high expression of IMPDH1 was closely associated with low therapeutic response and poor prognosis in tumor patients receiving immunotherapy.

**Figure 8:**
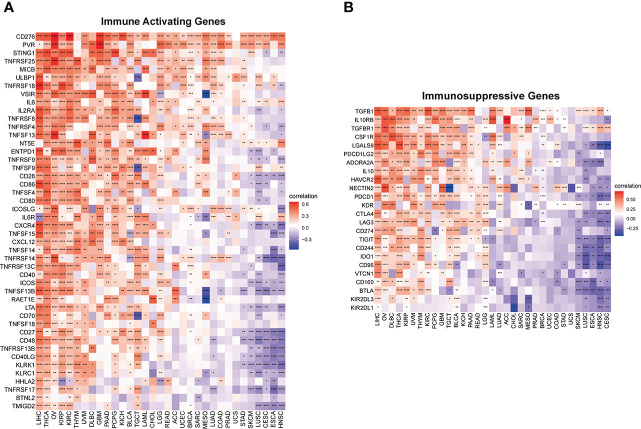
IMPDH1 is associated with immune activating and immunosuppressive genes. **(A)** Evaluation of the correlation of IMPDH1 expression with immune activating genes using TCGA database. **(B)** Correlation of IMPDH1 expression with immunosuppressive genes from TCGA database. *p < 0.05; **p < 0.01; ***p < 0.001 and ****p < 0.0001.

**Figure 9:**
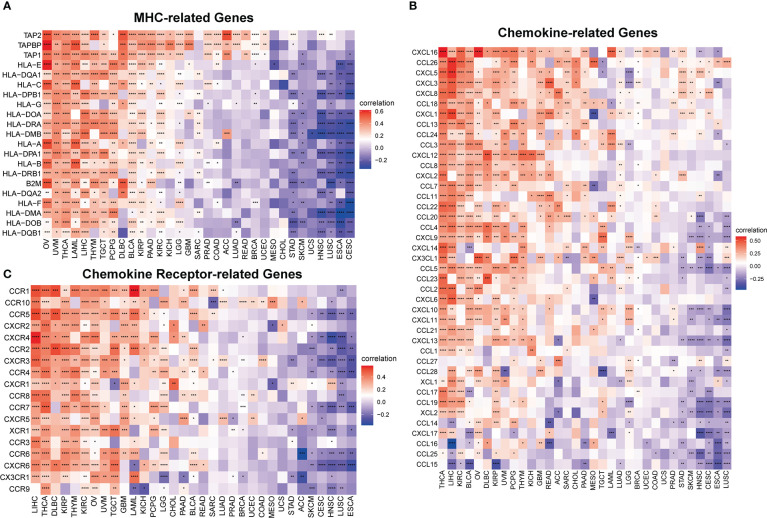
IMPDH1 is associated with immune progress-related genes. **(A)** Relationship between IMPDH1 and MHC-related genes from TCGA database. **(B)** Relationship between IMPDH1 and Chemokine-related genes from TCGA database. **(C)** Relationship between IMPDH1 and Chemokine Receptor-related genes from TCGA database. *p < 0.05; **p < 0.01; ***p < 0.001 and ****p < 0.0001.

Furthermore, to comprehensively assess the role of IMPDH1 in the immune system, we also evaluated the relationship between IMPDH1 and some immune-related genes. Firstly, we analyzed the association between IMPDH1 and a gene-set capable of activating tumor immunity in pan-cancer (**Figure 8A**), and a gene-set capable of suppressing tumor immunity from TCGA database (**Figure 8B**). Interestingly, IMPDH1 was not only closely related to immune activation genes, but also strongly associated with immunosuppressive genes. Moreover, in some types of tumors such as LIHC, OV, THCA, There was positive association of IMPDH1 with both immune activating and immunosuppressive genes. Of course, in some types of tumors such as HNSC, CESC, ESCA, IMPDH1 was negatively related to both immune activating, and immunosuppressive genes, which represented the complexity of IMPDH1 in tumor immune regulation. Immune-related genes also include genes involved in immune progress. Hence, the analysis of correlation between MHC-related genes and IMPDH1 was conducted, showed a strong relationship (**Figure 9A**). In addition, we also examined the corresponding genes of Chemokine and Chemokine receptor pathways. Even though the range of tumor types with statistical differences was not as wide as other immune-related genes, they still accounted for half the proportion and were important (**Figures 9B, C**).

### 3.4 High expression of IMPDH1 correlates with hypomethylation

**Figure 10:**
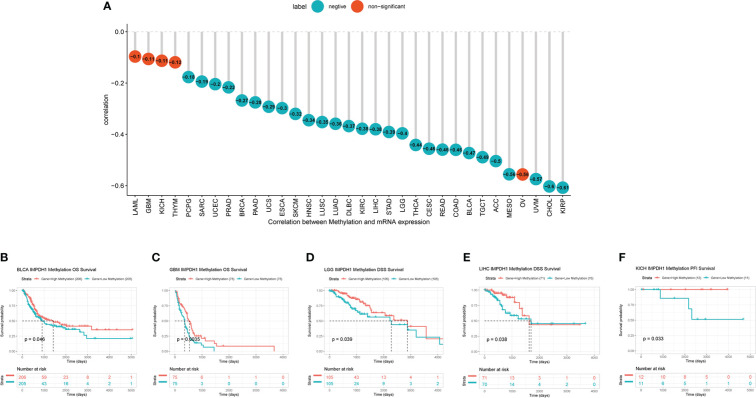
High expression of IMPDH1 correlates with DNA hypomethylation and methylation-related survival. **(A)** Evaluation of the correlation between IMPDH1 expression and methylation. Cyan circles represent a negative correlation and red circles represent non-significant results. The number in the circle represents the correlation coefficient. **(B, C)** The association of IMPDH1 expression with methylation-related OS of BLCA and GBM patients. **(D, E)** The association of IMPDH1 expression with methylation-related DSS of LGG and LIHC patients. **(F)** The association of IMPDH1 expression with methylation-related PFI of KICH patients.

IMPDH1 was highly expressed in most tumors, but we also wondered what caused it. As well known that DNA methylation, as a common covalent modification regulation, was able to control gene expression. Therefore, we analyzed the correlation of methylation level with IMPDH1 expression in pan-cancer, showing negative correlation in 28 kinds of tumors (**Figure 10A**). Additionally, some tumors, like BLCA, GBM, LGG, LIHC, KICH also had prognostic differences related to the level of IMPDH1 methylation (**Figures 10B-F**).

### 3.5 IMPDH1 is associated with the infiltration and several functions of immune cells in HCC

**Figure 11:**
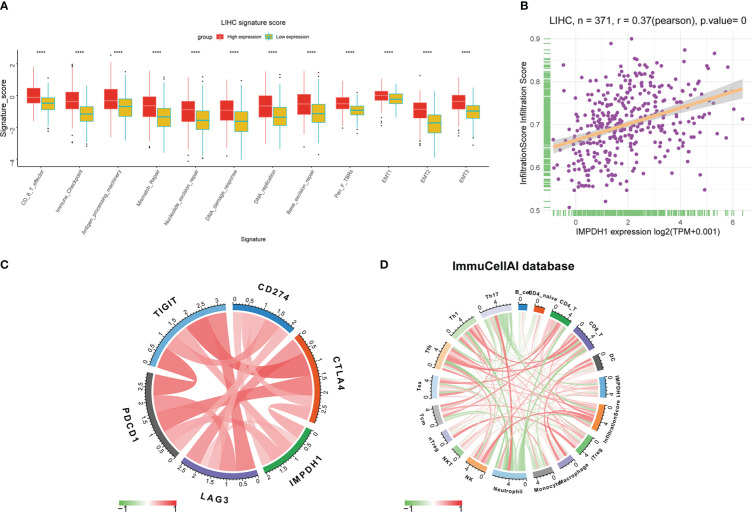
IMPDH1 is associated with immune cells infiltration and immune checkpoint in HCC. **(A)** Analysis of the relationship between IMPDH1 expression and LIHC signature score. **(B)** The correlation of IMPDH1 expression and infiltration score in LIHC. **(C)** The correlation between IMPDH1 expression and 5 types of immune checkpoint. **(D)** The correlation between IMPDH1 expression and immune cells infiltration in tumor immune microenvironment. Red represents a positive correlation, and green is negative represents a negative correlation. The darker the color, the stronger the correlation. ****p < 0.0001.

By analyzing the signature score in tumor microenvironment of HCC, it could be clearly seen that the infiltrating CD8^+^T lymphocytes and immune checkpoint in the tumor microenvironment with high expression of IMPDH1 were significantly increased (**Figure 11A**), and the infiltration score also increased due to the high expression of IMPDH1 (**Figure 11B**). PD-1, TIGIT, and CTLA4 were closely related to IMPDH1 among several common clinical immune checkpoints: PD-1, PD-L1, CTLA4, TIGIT, LAG3 (**Figure 11C**), which might affect immunotherapy sensitivity. In HCC, IMPDH1 was correlated with numerous immune cells indicated by ImmuCellAI database (**Figure 11D**).

**Figure 12:**
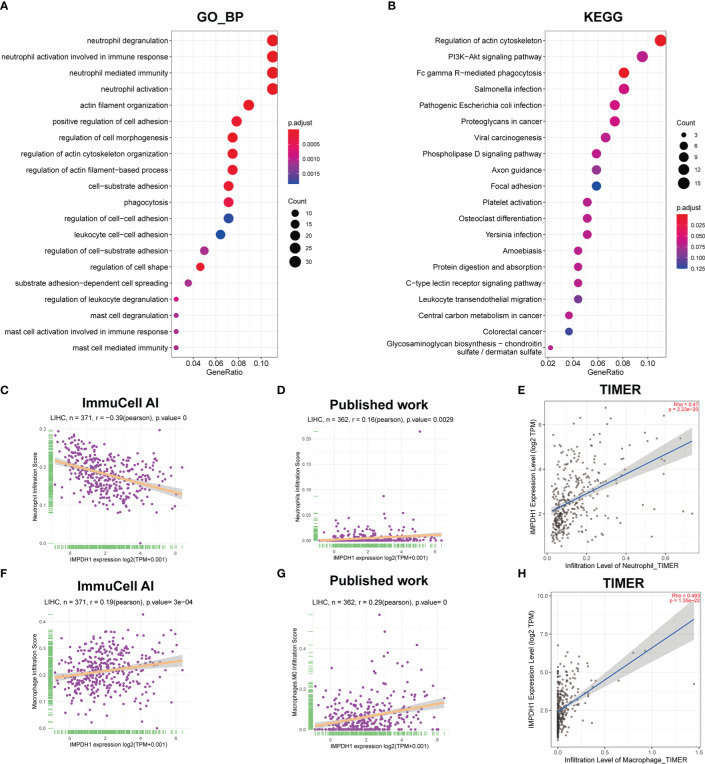
IMPDH1 is related to neutrophils and macrophages associated innate immunity in HCC. **(A)** GO_BP analysis of IMPDH1. **(B)** KEGG analysis of IMPDH1. **(C)** The association of IMPDH1 expression with neutrophils infiltration in LIHC using ImmuCell AI database. **(D)** The association of IMPDH1 expression with neutrophils infiltration in LIHC using published study data. **(E)** The association of IMPDH1 expression with neutrophils infiltration in LIHC using TIMER2 database. **(F)** The association of IMPDH1 expression with macrophage infiltration in LIHC using ImmuCell AI database. **(G)** The association of IMPDH1 expression with macrophage M0 infiltration in LIHC using published study data. **(H)** The association of IMPDH1 expression with macrophage infiltration in LIHC using TIMER2 database. The darker the red color of the circle, the smaller the p value, and the darker the blue color, the larger the p value. The larger the circle area, the more genes contained in the enriched pathway.

In addition to CD8+T cells, IMPDH1 expression also had a great influence on the infiltration of other immune cells. We performed Gene Ontology (**Figure 12A**) and Kyoto Encylopedia of Genes and Genomes (KEGG) analysis (**Figure 12B**), and the results suggested that IMPDH1 was involved in neutrophil degranulation, neutrophil-associated innate immunity, and FC gamma R-mediated phagocytosis. We then analyzed the association of IMPDH1 with neutrophils (**Figures 12C-E**) and macrophage infiltration (**Figures 12F-H**) in HCC using the ImmuCellAI database, TIMER database, and datasets from previously published studies (26, 27). It was found that a strong correlation between these two types of immune cells and the infiltration of IMPDH1.

### 3.6 The influence on immune microenvironment by IMPDH1 may be related to the modification of m-6-A in HCC

**Figure 13:**
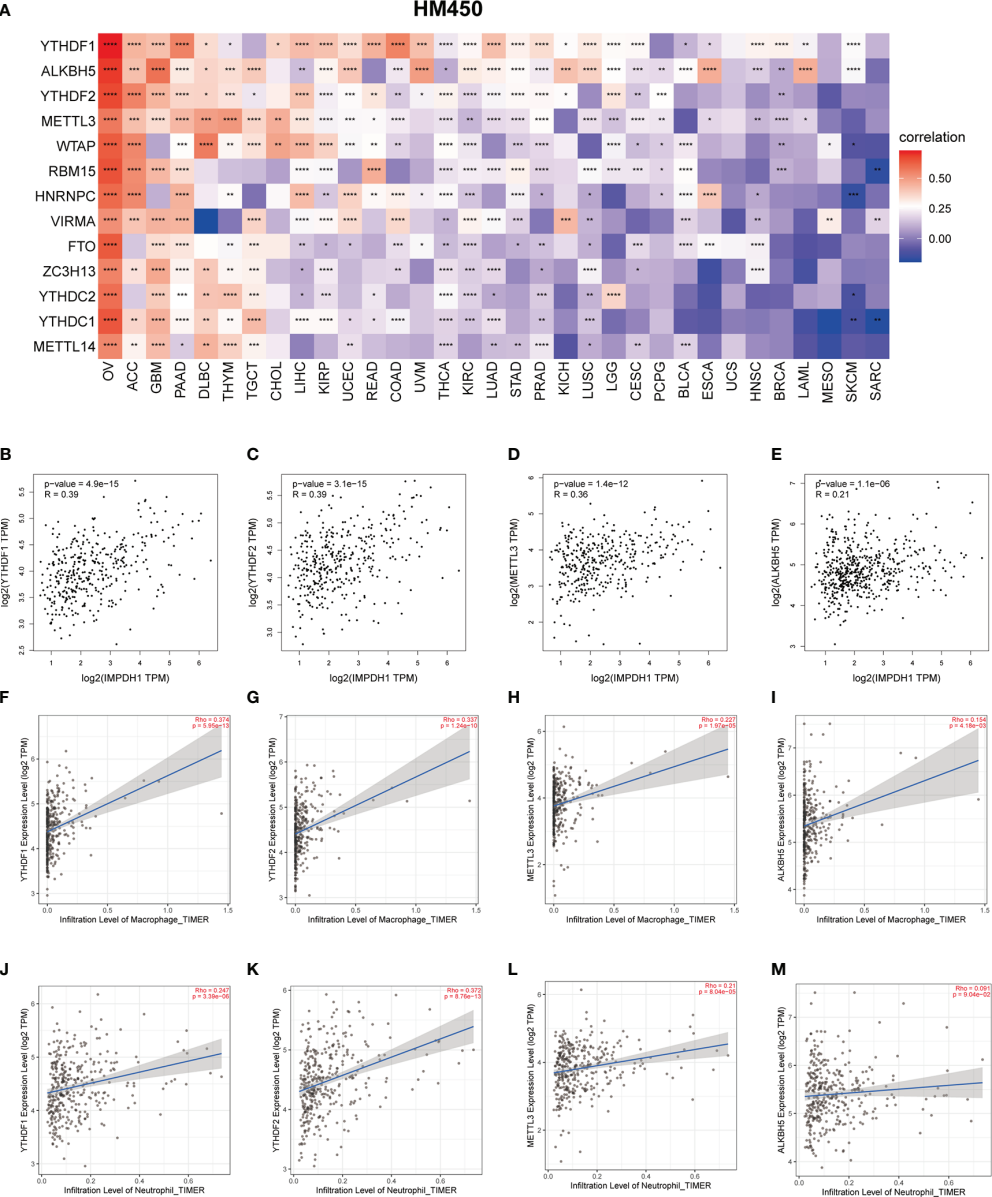
M-6-A modification may be involved in influencing neutrophil and macrophage infiltration related to IMPDH1 expression in HCC. **(A)** Relationship between IMPDH1 and genes encoding m-6-A modified proteins in pan-cancer from TCGA database. **(B–E)** The correlation between IMPDH1 and genes encoding m-6-A modified proteins: YTHDF1, YTHDF2, METTL3, and ALKBH5 using GEPIA2 database. **(F–I)** The correlation of genes encoding m-6-A modified proteins: YTHDF1, YTHDF2, METTL3, ALKBH5 with macrophages infiltration using TIMER2 database. **(J-M)** The correlation of genes encoding m-6-A modified proteins: YTHDF1, YTHDF2, METTL3, ALKBH5 with neutrophils infiltration using TIMER2 database. *p < 0.05; **p < 0.01; ***p < 0.001 and ****p < 0.0001.

**Figure 14:**
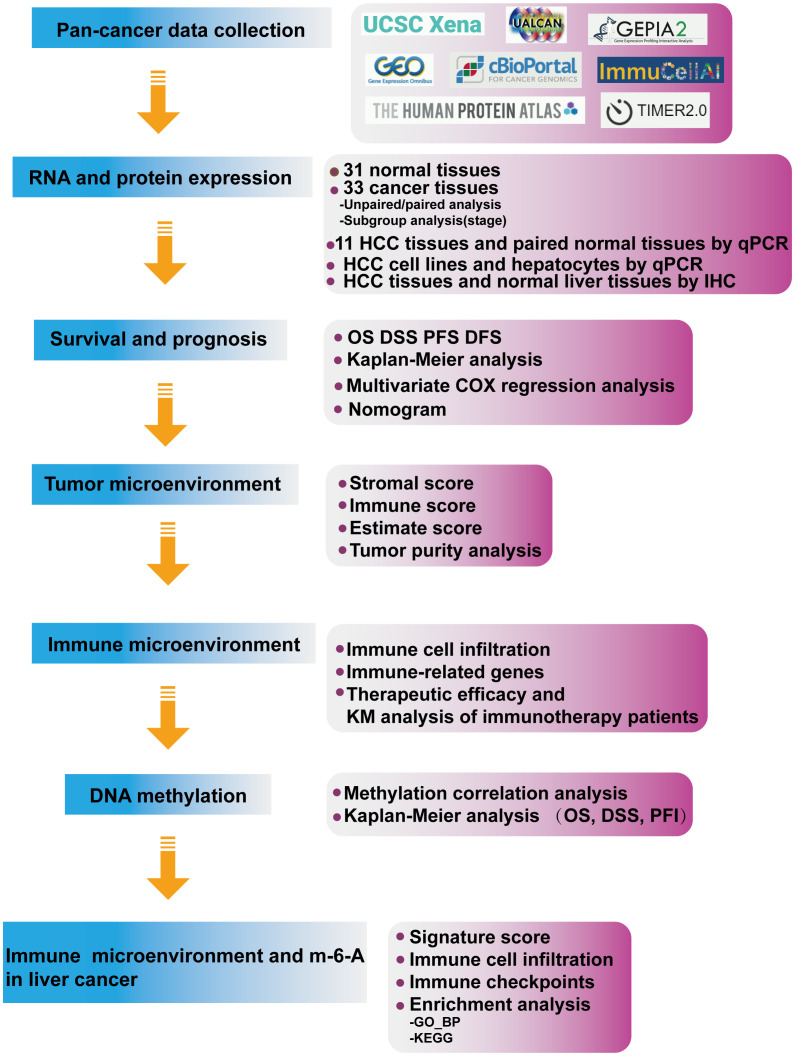
Flow chart.

M-6-A was the most common methylation modification and was highly influential in tumor development. We evaluated m-6-A modified protein, which showed IMPDH1 was closely related to m-6-A related regulatory proteins in pan-cancer (**Figure 13A**), and we focused on evaluating the association of IMPDH1 with the top four proteins: YTHDF1, ALKBH5, YTHDF2, METTL3 in HCC (**Figures 13B-E**), indicating a strong relationship. Then we analyzed the correlation of such four proteins with macrophage, and neutrophil. It could be found that m-6-A modified proteins was closely associated with the infiltration of macrophage and neutrophil (**Figures 13F-M**). Finally, we utilized a flow chart to show the research route of IMPDH1 in pan-cancer more intuitively (**Figure 14**).

## 4 Discussion

Cancer is a major concern as one of the leading cause of death worldwide (28). Surgery, radiotherapy and chemotherapy have always been the mainstay treatments for solid tumors. However, recurrence or metastasis often occurs, resulting in poor efficacy (29). Until the advent of immunotherapy, it was rare in the history of cancer treatment that a single treatment was effective for multiple diseases (30). Actually, due to the clinical success of immune checkpoint blockers, in 2013, science named “Cancer Immunotherapy” as the Breakthrough of the Year (31). Immunotherapy has been shown to work well in tumors with high tumor mutational burden (TMB) (32, 33), but due to factors such as the modification for the structure of the therapeutic drugs, the patient’s constitution and improper medication, some patients develop immune resistance and low response rate to immunotherapy (34–36). Sensitizing immune checkpoint inhibitors by activating the immune microenvironment and affecting immune checkpoints may be a potential therapy for cancer patients.

Through bioinformatics analysis, we found that IMPDH1 was in a state of high expression in many tumors, and the cancer tissues were mostly higher than the paired adjacent tissues. Relatively high expression in advanced tumors (Stages 3/4), indicating that IMPDH1 was involved in the tumor progression, the advanced stage of the tumors, especially. In IMPDH family, only IMPDH1 is significantly overexpressed in renal clear cell carcinoma, which is consistent with our analysis (23). Generally, protein makes a more direct and obvious functional influence than mRNA in organisms. We not only analyzed the mRNA expression of IMPDH1, but also assessed that the protein expression of IMPDH1 was also up-regulated in various tumors. Of course, after verification by qRT-PCR and IHC, it was found that the expression of IMPDH1 in HCC tissues and cell lines was consistent with the results of public datasets. Patients with high IMPDH1 expression typically usually had a relatively poor prognosis. At present, some studies use gene set, RNA set model to predict the occurrence of disease and the prognosis of patients. Through integrating 12 differentially expressed miRNAs, it may affect the proliferation, invasion and metastasis of endometrial cancer, and then predict the prognosis of patients (37). Likewise, as an important component of the ferroptosis-mediated metabolic change gene set, IMPDH1 is found to be a risk factor in the risk score model for predicting OS of HCC patients (38). Moreover, novel 5-gene signature-related to lipid metabolism including IMPHD1 can also be used as independent risk factor for bladder cancer patients (39), suggesting that IMPDH1-targeted agents might be useful in the treatment of tumors. The correlation between IMPDH1 and Immune score, Stromal score, Tumor purity in the tumor microenvironment suggested that more than one-third of types of cancers in pan-cancer are positively related to Estimate Score, and about one-third are negatively correlated with Estimate Score. Of course, it also has the potential to be used in combination with other first-line immunotherapy agents. Just as CTLA4 antagonists alone can only restore PD-1^+^CTLA-4^+^ TILs response to tumor antigens, but not CTLA4^-^ TILs (40), single immune checkpoint blockade targets a limited subset of CD8^+^T cells, while dual or multiple immunotherapies lead to progressively better outcomes. Subsequently, we used a variety of sources to analyze the correlation between IMPDH1 and immune cells in TME. Immune cells are able to regulate and predict the process of immunotherapy through related hub genes, including a variety of immune cells in the process of tumor immunity, such as CD8^+^T cells, tumor-associated macrophages, natural killer cells, eosinophils, etc (41–43). The strongest relationship suggested that IMPDH1 might play an immunoregulatory role through CD8^+^T lymphocytes and monocyte-macrophages. In addition, IMPDH1 was positively related to immune-related genes, no matter whether the genes were associated with activation or suppression of immunity, which suggested a complex state of IMPDH1 against tumor immunity. Because there is a feedback mechanism in the local tumor environment, and related anti-tumor inflammation often recruits more immunosuppressive cells, resulting in simultaneous infiltration of immunosuppressive cells and immune activating cells in the TIME (44). Similarly, the positive correlation between immunosuppressive factors and immune-stimulatory factors also indicates that the process of immune activation and immunosuppression occur simultaneously. In addition, MHC-related molecules that significantly influence CD4+ and CD8+ T-lymphocytes antigen presentation (45), as well as chemokine and chemokine receptor-related pathway genes that mediate immune cell transport and immunotherapy resistance (46, 47), affect immunity, which also had an obvious relationship with IMPDH1. At present, tumor immunotherapy not stops at the treatment plan for immune checkpoints, but also has therapeutic strategies that directly act on the tumor immune microenvironment, and the way forward still needs to be explored (48).

Moreover, we have demonstrated that in a variety of tumors, the expression of IMPDH1 attenuated the efficacy of immunotherapy and reduced the survival of tumor patients through using different databases, which was more convincing that as IMPDH1, as an undesirable immune molecule, its inhibitors might be used as sensitizers in cancer immunotherapy to benefit patients. Additionally, a large number of previous studies have shown that gene expression level is closely related to DNA methylation, and integrated gene-gene graph has even been constructed to reflect the gene methylation characteristics that combinedly affect the gene expression profile (49). To investigate the molecular mechanism of IMPDH1 overexpression in tumors, we performed a series of correlation analyses between IMPDH1 and its DNA methylation level. In the vast majority of tumors, the expression level of IMPDH1 was negatively correlated with methylation level, which partly explained it’s high expression in tumors. In addition, the OS, DSS and PFS of some tumor patients were also closely related to IMPDH1 methylation level.

Exited some opposite association might occur among different tumors due to tumor heterogeneity. Therefore, it is more appropriate to discuss different tumors separately. Since the significant results of HCC in the pan-cancer study of IMPDH1, and HCC is the fourth leading cause of cancer-related mortality in China (50), with large patient population, which suggest HCC might be the most representative cancer in pan-cancer for IMPDH1 study. Thus we mainly discussed and verified IMPDH1 in HCC. Analysis of the correlation between HCC signature score, infiltration score, various immune cell infiltration, important immune checkpoints and IMPDH1 showed that high expression of IMPDH1 may increase CD8+T cell infiltration and the expression of immune checkpoints such as PD-1 and CTLA4, while targeting IMPDH1 might contribute to immunotherapy. Several studies have found that PD1^Hi^CD8^+^T cells are often in a exhausted status and secrete low levels of cytotoxic molecules, and such HCC patients are usually have a poor prognosis (51, 52). Additionally, GO and KEGG gene enrichment analyses suggested that IMPDH1 was associated with neutrophil-related innate immunity and FC gamma R mediated phagocytosis in HCC. As innate immune cells, neutrophils promotes tumor progression by regulating the expression of various inflammatory factors and influencing phagocytosis (53). Targeting or manipulating neutrophils may gain clinical benefits and are expected to be combined with immunotherapy (54, 55). Genes, non-coding RNAs, and proteins can regulate key pathways or molecular modifications to influence immune components in the tumor microenvironment and thus affect the therapeutic effect of tumors patients. Lncrna00654-ninl regulatory axis affects the prognosis and immunotherapy of patients with diffuse large B-cell lymphoma by regulating NF-κB pathway and neutrophils. Therefore, we discussed how IMPDH1 regulated the immune microenvironment of HCC. Enzyme proteins related to m-6-A modification have been reported to be related to immunotherapy in many previous studies. High expression of YTHDF1 and METTL3 has been reported to be associated with lower OS in cancer patients (56, 57), and YTHDF2, YTHDF1 and METTL3 were proved to be associated with sorafenib treatment and anti-PD-1 immunotherapy response in HCC (58). In pan-cancer research, there was a strong correlation between IMPDH1 and m-6-A modified protein. At least two of the top 4 m-6-A modified protein-encoding genes were positively correlated with tumor-associated macrophages and neutrophils in HCC, displaying it is possible that IMPDH1 remodeled TIME through m-6-A modified proteins.

## 5 Conclusion

IMPDH1 is highly expressed in a variety of cancers and usually predicts a poor prognosis. The low DNA methylation level of IMPDH1 is likely to be the mechanism of the high expression of IMPDH1 in tumors. IMPDH1 affects a variety of immune cells in tumor immune microenvironment and is closely related to multiple immune-related pathways. More importantly, the expression level of IMPDH1 makes a considerable contribution to the treatment response and prognosis of cancer patients receiving immunotherapy. Furthermore, IMPDH1 may remodel the HCC immune microenvironment through m-6-A modified proteins. In summary, IMPDH1 is highly expressed in tumor tissues and influences the modification of immune microenvironment. As a potential therapeutic target, IMPDH1 may play a critical role in tumor immunotherapy. For the future research, it is more necessary to focus on HCC or other specific tumors. Utilizing single-cell sequencing analysis and experiments *in vivo* and *in vitro* to find and verify the specific cell subtypes and molecular mechanisms of IMPDH1’s immunoregulation in the tumor microenvironment. In addition, the design of specific IMPDH1 inhibitors and related clinical trials will certainly contribute to the development of tumor immunotherapy.

## Data availability statement

The original contributions presented in the study are included in the article/**Supplementary Material**. Further inquiries can be directed to the corresponding author.

## Ethics statement

The studies involving human participants were reviewed and approved by the Institutional Review Committee of Nanfang Hospital, Southern Medical University, Guangdong Province, China. The patients/participants provided their written informed consent to participate in this study.

## Author contributions

LL, WZ, CL, and XZ designed the study. WZ, CL, and XZ performed data analysis. WZ, CL wrote the manuscript and helped with validation. All authors interpreted the results and read and approved the final version of the manuscript.

## Supplementary material

Please refer to the supplementary documents for details.
