# Supplementary figures and images for: IMPDH1, a prognostic biomarker and immunotherapy target that correlates with tumor immune microenvironment in pan-cancer and hepatocellular carcinoma
Source: Front Immunol. 2022 Dec 22;13:983490. doi: 10.3389/fimmu.2022.983490 (PMC9813230; doi:10.3389/fimmu.2022.983490)

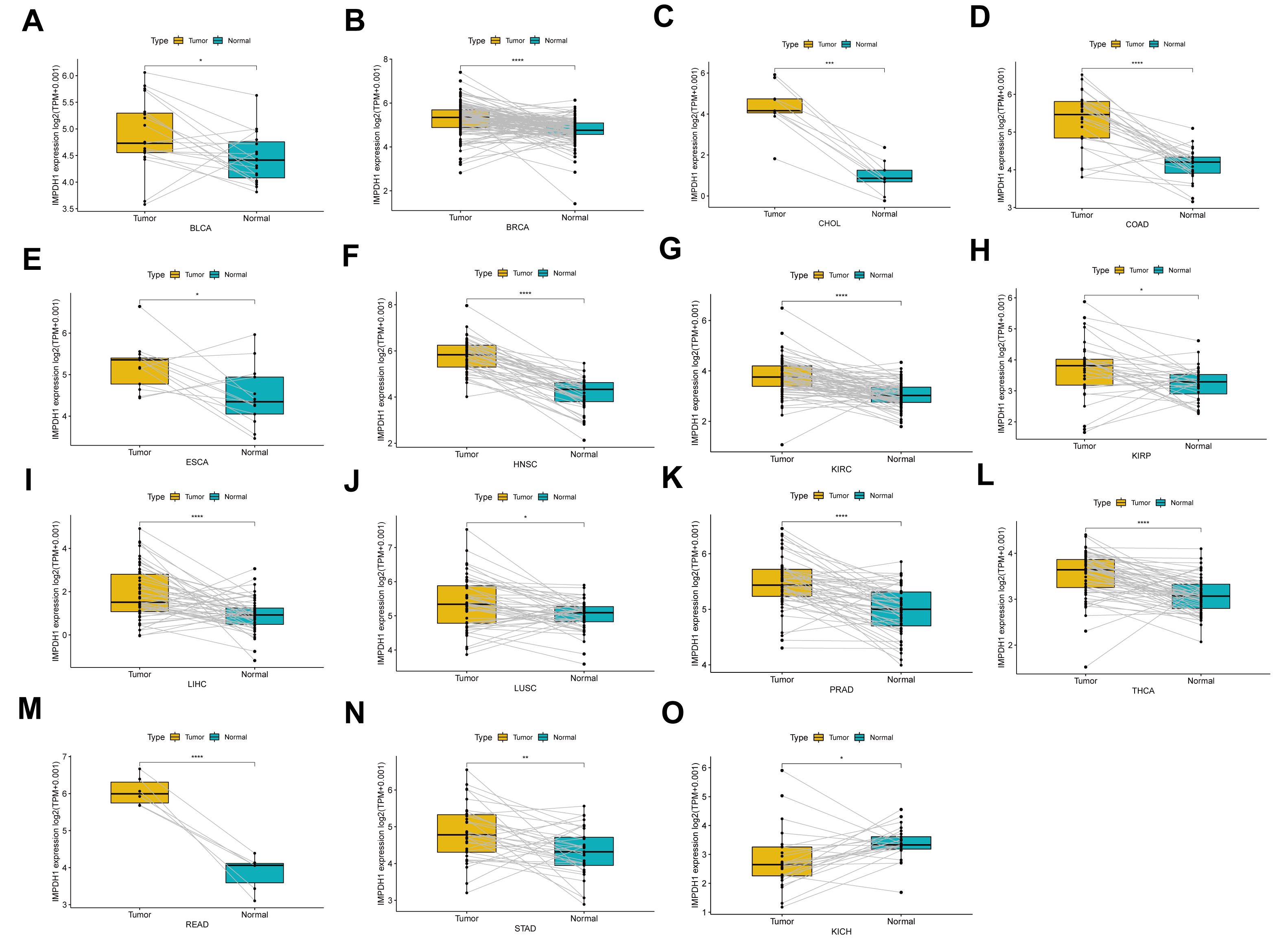

Supplement: Supplementary file 1 [file Image_1.tif]

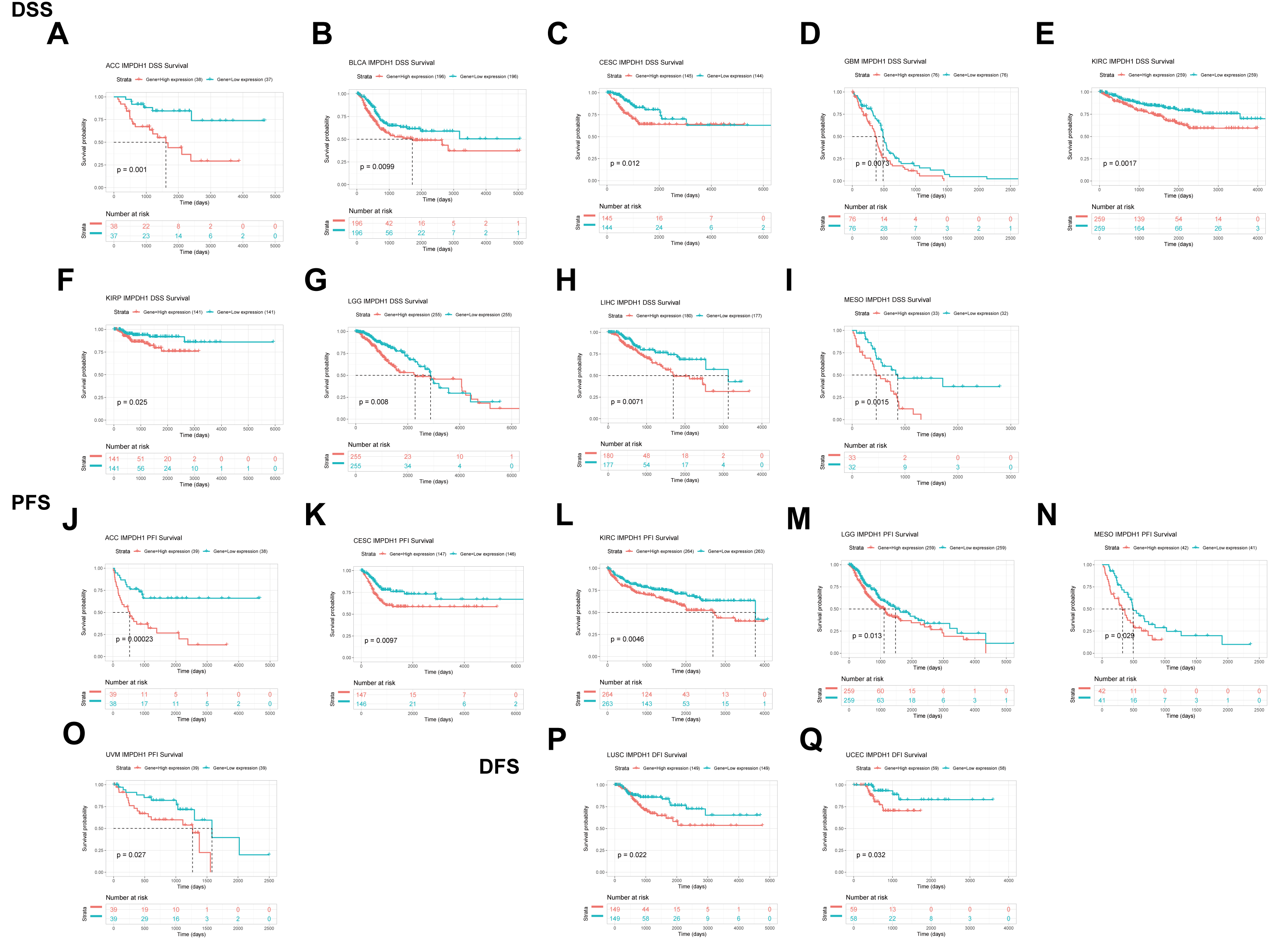

Supplement: Supplementary file 2 [file Image_2.tif]

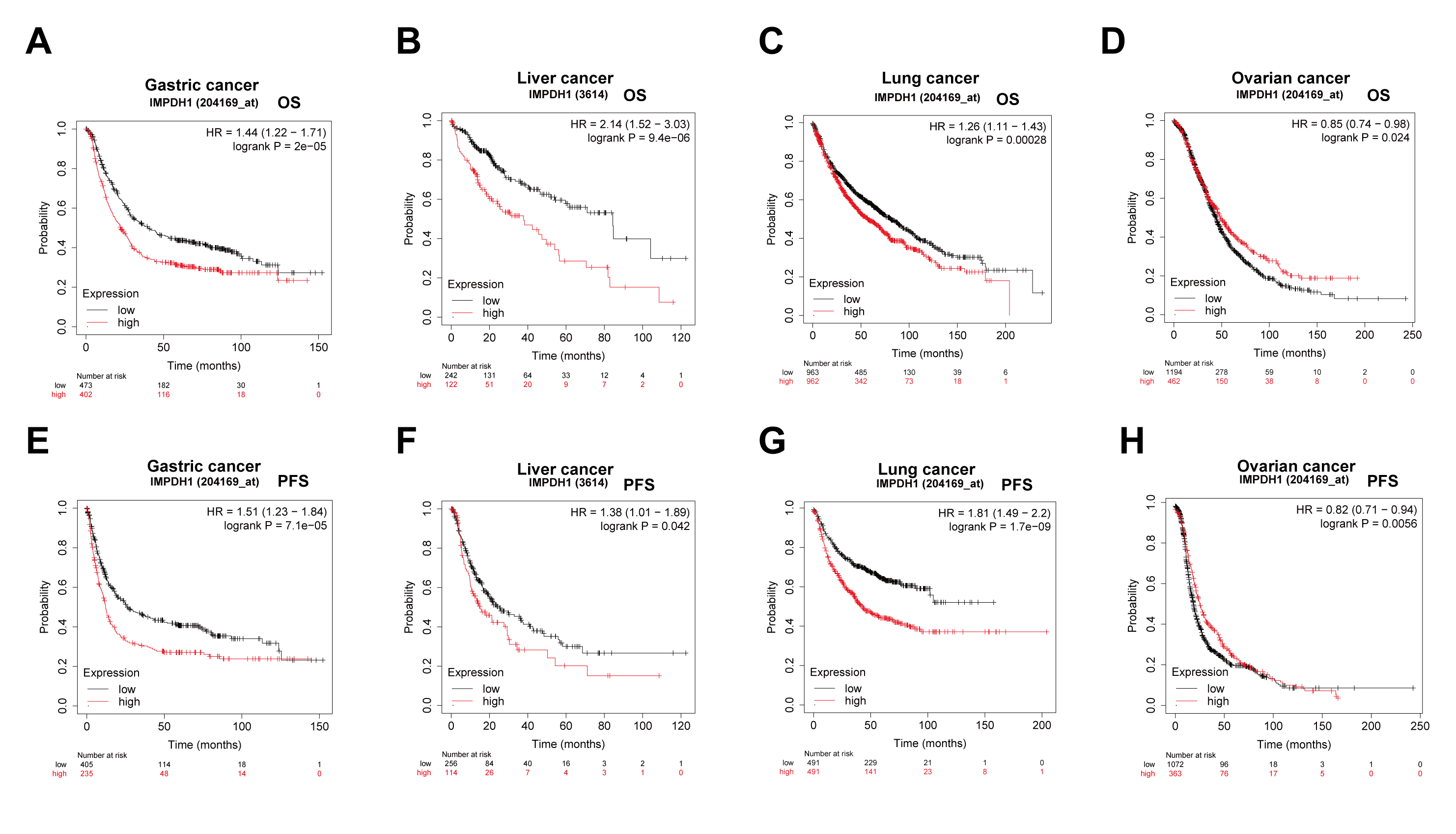

Supplement: Supplementary file 3 [file Image_3.tif]

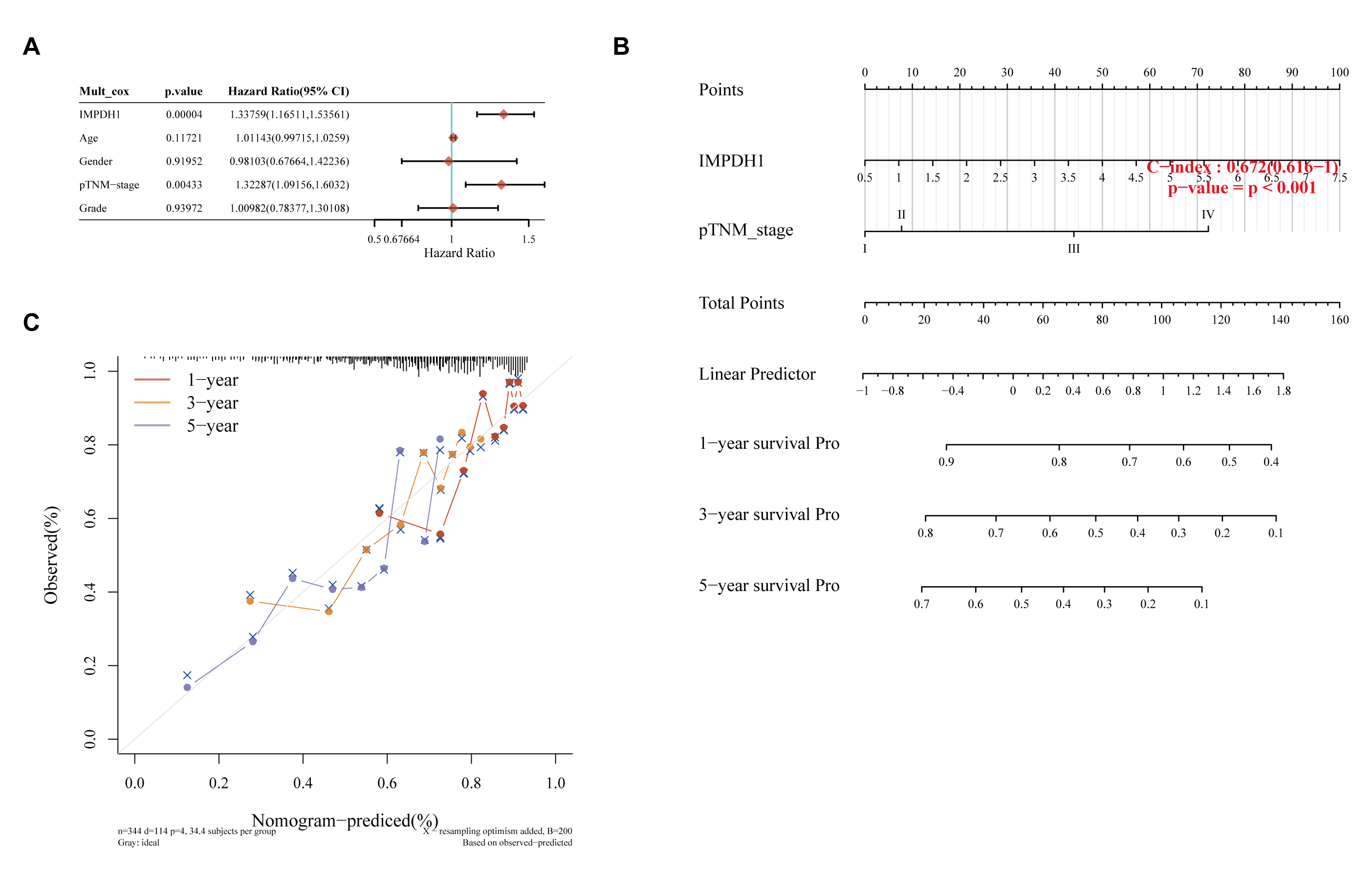

Supplement: Supplementary file 4 [file Image_4.tif]
